# β-Glucosidase Activity of *Lactiplantibacillus plantarum*: A Key Player in Food Fermentation and Human Health

**DOI:** 10.3390/foods14091451

**Published:** 2025-04-22

**Authors:** Gianluca Paventi, Catello Di Martino, Francesca Coppola, Massimo Iorizzo

**Affiliations:** 1 Department of Agricultural, Environmental and Food Sciences, University of Molise, Via De Sanctis, 86100 Campobasso, Italy; lello.dimartino@unimol.it; 2 Department of Agricultural Sciences, University of Naples “Federico II”, Portici, 80055 Naples, Italy; francesca.coppola2@unina.it

**Keywords:** lactobacilli, functional food, lactic acid bacteria, glycosides, aglycones

## Abstract

β-glucosidases are a relevant class of enzymes in the food industry due to their role in hydrolyzing different types of glycosidic bonds. This activity allows for formation of volatile compounds and release of bioactive aglycone compounds. In addition to endogenous β-glucosidase activity present in raw material, the function of β-glucosidases in fermenting microorganisms has been progressively clarified and increasingly appreciated. In this regard, several lactic acid bacteria, including *Lactiplantibacillus plantarum*, showed high β-glucosidase activity, which can be considered as a valid biotechnological resource in different food sectors. Here, we reviewed the huge literature in which the β-glucosidases of *L. plantarum* were shown to play a role, highlighting how their action results in enhancing the nutritional, sensory, and functional properties of fermented foods. To this aim, after a brief introduction of the main functions of these enzymes in several kingdoms, we critically analyzed the involvement of *L. plantarum* β-glucosidases in plant-based food production, with a particular insight for soy, cassava, and olive-fermented products, as well as in the production of both alcoholic and non-alcoholic beverages. We trust that the reports summarized here can be helpful in planning future research and innovative strategies to obtain pleasing, functional, and healthy foods.

## 1. Introduction

β-glucosidases are a class of exoglucosidase enzymes capable of acting on terminal non-reducing β-D-glucosyl residues by hydrolyzing the β-1,4 glycosidic bond of different glycoconjugates including glucosides, oligosaccharides, and 1-*O*-glucosyl esters, with the release of β-D-glucose [1]. Although in some limited cases β-*S*-glucosidase activity was also reported [2], these enzymes act on *O*-glycosylated substrates, releasing aglycone compounds. On the basis of their amino acid sequences, they are classified in families and clans that share a conserved catalytic mechanism, structure, and active site residues, but may vary in substrate specificity [3,4]. These enzymes are ubiquitous in nature and are found in all domains of living organisms—Archaea, Eubacteria, and Eukaryotes (fungi, plants, and animals, including humans) [5]. In these organisms, β-glucosidases play a significant role in various biological processes and functions including nutritional acquisition and ecological associations. However, most organisms utilize this enzyme for the hydrolysis of oligosaccharides to glucose, the most usable form of carbon. The β-glucosidases fall in the enzyme class EC 3.2.1.21. At present, 133 glycoside hydrolase (GH) families are listed in the frequently updated Carbohydrate Active enZYme (CAZY) database (http://www.cazy.org, accessed on 19 April 2025) [6]. So far, they have been classified into GH1, GH3, GH5, GH9, and GH30. Family GH1 includes β-glucosidases from archaebacteria, plants, and mammals, and family GH3 comprises β-glucosidases of some bacterial, mold, and yeast origin [7,8,9,10]; GH9 β-glucosidases are reported to differ in their mechanism of action with respect to GH1, GH3, and GH30 family members [10]. Structures and sequences for both GH1 and GH3 β-glucosidases were recently reviewed by Ouyang [11].

In plants, β-glucosidases perform a wide range of biological functions such as pathogen and insect resistance, microbial interactions, lignification, phytohormones activation, signaling mechanisms, cleavage of glycosylated flavonoids, fruit ripening, and pigment metabolism [5,12,13,14,15,16].

In humans, three native β-glucosidase enzymes have been identified: glucocerebrosidase, deficiency of which causes Gaucher’s disease; lactase phlorizin hydrolase, deficiency of which causes lactose intolerance; and β-glucosidase, a cytosolic enzyme of broad specificity that is abundant in the kidney, liver, and small intestine of mammals and plays a crucial role in the transport and/or digestion of dietary sugars [17,18,19].

In insects, β-glucosidases are mainly involved in cellobiose digestion, the breakdown of glucosinolates (glucosylated specialized metabolites) sequestered from host plants to form a dual-component defense system, and communication and recognition among sexual or social interactions. These functions have been found in different groups of insects, and they adapt to the system based on the plants they feed on [20,21].

The genetic diversity and expression of β-glucosidase-producing microorganisms were studied in different habitats, including food, soil, cow dung and compost, and marine environments [22,23,24,25,26,27]. In bacteria and fungi, β-glucosidase is a crucial element of the microbial cellulose multienzyme complex since it is responsible for the regulation of the entire cellulose hydrolysis process by easing cellobiose-mediated suppression and producing the final product glucose [22,28]. The fungal species *Aspergillus niger* is the major source of commercial β-glucosidase; however, this enzyme has been produced, purified, and characterized from other mold genus (e.g., Trichoderma, Aspergillus, Penicillium, Fusarium, etc.), with the majority of which belonging to GH3 as cited in [29].

In addition, the β-glucosidase has been identified, purified, and characterized from many bacteria (e.g., *Clostridium*, *Bacillus*, *Paenibacillus*, *Bifidobacterium*, etc.) including some Lactic Acid Bacteria (LAB), as reported in previous reviews and research articles [30,31,32,33]. In particular, the β-glucosidase produced by LABs plays an important role in the performance of these microorganisms in food fermentation or during interaction with their hosts in the intestinal environment, as reported by Michlmayr et al. in a previous review [30]. For example, they have the potential to improve the flavor and aroma of alcoholic (e.g., wine and beer) and non-alcoholic beverages (e.g., teas and juices) by releasing aromatic compounds from flavorless glycosides. Microbial β-glucosidase is also used to hydrolyze isoflavonic glycosides (e.g., soybean products), as well as oleuropein, thus reducing the bitterness of table olives [7].

During food fermentation or by interaction with their hosts, several LAB can provide β-glucosidase activities to increase the bioavailability of metabolites that improve the host health, such as plant phenolic compounds, which are usually glycosylated in their dietary format, and therefore are less bioavailable than the aglycone forms [34,35,36]. A known example includes the soy isoflavones, which can be released from their glycosylated precursors by some LAB β-glucosidases during soy fermentation [37]. The β-glucosidase activities from LAB may also have implications for food security. Cassava contains high concentrations of the toxic cyanogenic glucoside linamarin, and LAB contributes to the degradation of linamarin by β-glucosidase activities [7].

On the other hand, it is known that the mycotoxin deoxynivalenol is not toxic in its glycosylated form (deoxynivalenol-3-glucoside), but it can be activated by a LAB β-glucosidase [38].

Given the importance of LAB β-glucosidases, considerable efforts have been focused to increase our knowledge on these enzymes, which usually show a broad specificity [30].

Among the LAB able to produce β-glycosidases, *Lactiplantibacillus plantarum* (formerly *Lactobacillus plantarum*) represents an important member. This heterofermentative species is also known for its high adaptability to many different conditions, since it has been isolated from various ecological niches including milk, fruit, cereal crops, vegetables, bee bread, and fresh meat [39,40,41,42,43], as well as fermented foods [44,45]. Moreover, this species is widely diffused into the gastrointestinal tract of animals; several studies, in fact, showed that it colonizes the digestive system of insects [46,47,48], fish [49], and mammals, including humans [50]. The inclusion of *L. plantarum* in both QPS (Qualified Presumption of Safety) and GRAS (Generally Recognised as Safe) lists [51,52], together with the many intrinsic properties of this species, led to the proposal of numerous *L. plantarum* strains as animal and human probiotics [53,54].

*L. plantarum* is widely used as a starter culture in the fermentation of raw materials of plant and animal origin, where it contributes to enhancing the sensorial quality and shelf-life of fermented products. Some *L. plantarum* strains also increase the functional properties of various fermented foods by producing a variety of bioactive compounds [55,56].

The present review aims to provide an overview of the role of the activity of β-glycosidase produced by *L. plantarum* as a valid biotechnological resource in different food sectors. In particular, following a general description of the role/s of β-glycosidase in plant fermented foods, we critically analyzed the involvement of *L. plantarum* β-glucosidases in plant-based food production, with a particular insight for soy-, cassava-, and olive-fermented products, as well as in the production of both alcoholic and non-alcoholic beverages. Despite β-glycosidases reported to be potentially involved also in thioglycohydrolase activity [2], the main function in *L. plantarum*-fermented foods can be restricted to its action on *O*-glycosylated compounds as specificized in the subsequent paragraphs.

## 2. Roles of β-Glucosidases in Fermented Plant-Based Foods

The natural chemicals of fruit include volatile compounds, both in free and bound form, which occur primarily as glycoconjugates of sugar and an aglycone [57,58]. The sugar moiety includes glucose or a disaccharide, while the aglycone part of glycosides is often represented by monoterpenes, C13-norisoprenoids, benzene derivatives, and long-chain aliphatic alcohols [59,60,61,62]. Therefore, the hydrolysis of odorless glycosylated compounds can make an important contribution to improve the flavor of fruit juices and derived beverages [57]. In this regard, β-glucosidases play a major role since they hydrolyze glycosidic bonds occurring in aroma precursors thus promoting the release of free volatile compounds, as reviewed in [58].

Although endogenous β-glucosidases are present in fruits such as grapes, their activity is insufficient due to low stability under juice processing and winemaking conditions. In fact, the optimal pH levels at which plant glucosidases are most active generally range from 4.0 to 6.0; therefore, in the low pH of fruit juices, only limited activity of most glycosidases has been observed [63,64].

Due to this limited action of plant endogenous glycosidases, a large proportion of the aroma compounds in juices remain inactive, in a glycosidically bound form [65]. Research has therefore focused on finding exogenous sources of glucoside hydrolases, which can be used in the production of juices and wines [65,66].

Several procedures can be used to enhance wine aroma by releasing aroma compounds from glycosidic precursors, including acid or enzymatic hydrolysis. Acid hydrolysis causes rearrangements in the aglycone structure with the formation of undesirable flavors, while enzymatic hydrolysis specifically cleaves the glycosidic linkage without altering the aglycone structure [67].

**Figure 1:**
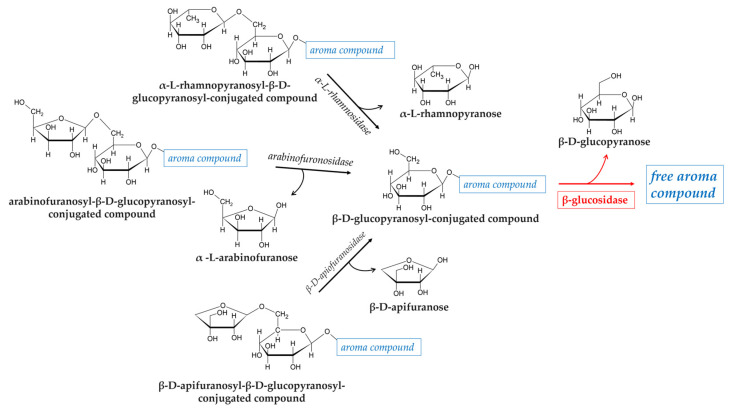
Release of free aroma compounds from glycosidically bound precursors by β-glucosidase activity. Representation of enzymatic activities involved in the release of the volatile aglycone component from three different disaccharide-bound aroma compounds.

Glycosidic precursors in fruits can be found as D-glucopyranosides in which the volatile aglycone is linked to a single D-glucopyranose by a β-glycoside bond. They can also occur as disaccharides, in which the D-glucopyranose is combined with a second sugar molecule such as α-L-arabinofuranose, α-L-rhamnopyranose, or α-L-apiofuranose (Figure 1).

The enzymatic hydrolysis of glycosidically bound aroma compounds occurs in two steps and involves different exoglycosidases depending on the sugar moieties of the substrates. For example, in the presence of rhamnose or apiose: first, a α-L-rhamnosidase or a β-D-apiofuranosidase cleaves the (1-6)-glycosidic linkage, and then, the flavor compounds are liberated from the monoglucosides by the action of a β-glucosidase. A hydrolysis scheme of glycosidic aroma precursors is shown in Figure 1.

Therefore, much attention has been attracted to the flavor enhancement of juices or wines through the hydrolysis of the glycoside aroma precursors using microbial β-glucosidases from mold, yeast, and LAB [68,69,70,71].

Phenolics, including flavonoids, widely distributed in plants, have received much attention and were recognized as the most abundant antioxidants in the human diet [72]. Increased antioxidative activity in fermented plant-based foods is primarily due to an increase in the amounts of phenolic compounds and flavonoid aglycones during fermentation, which is the result of a microbial hydrolysis activity [73].

Flavonoids are the largest class of polyphenols that can be further categorized into several subgroups including flavonols and anthocyanins, both of which are naturally distributed in plant foods as glycosides containing single or multiple sugar moieties. Flavonoid aglycones are generally more bioavailable than their respective glycosides [74].

Several studies have shown that flavonoid aglycones’ content in plant-based foods can increase after fermentation due to the microbial β-glucosidase. Therefore, fermentation by LAB possessing this specific enzymatic activity is an effective strategy to increase the bioavailability of natural antioxidants present in fermented plant-based products [75]. In this regard, the selection and use of probiotic starters, besides the production of fermented dairy products [76], represents an important biotechnological tool to be applied more widely, especially in the field of plant-derived fermented products [42]. Some LAB and bifidobacteria that possess β-glucosidase were proposed as a biotechnological resource in various fermented plant-based foods, as shown in Table 1.

### 2.1. Soymilk and Soybean Products

Isoflavones, which are produced almost exclusively by plants of the family Fabaceae, most often occur as glycosyl groups in plants. These compounds are found in plant sources mainly as O-glycosides, frequently bound to glucose, but also to other sugars such as galactose, rhamnose, arabinose, and xylose [102].

The biological activity of isoflavones has been well reported [103,104]. These compounds, known as phytoestrogens, are known to reduce the incidence of hormone-dependent steroid cancers such as breast, prostate, and colon cancer [105]. In addition, isoflavones have been shown to help prevent and treat several aging-related dysfunctions and diseases, including neurodegenerative disorders, osteoporosis, metabolic and cardiovascular diseases, and menopausal symptoms [106].

**Figure 2:**
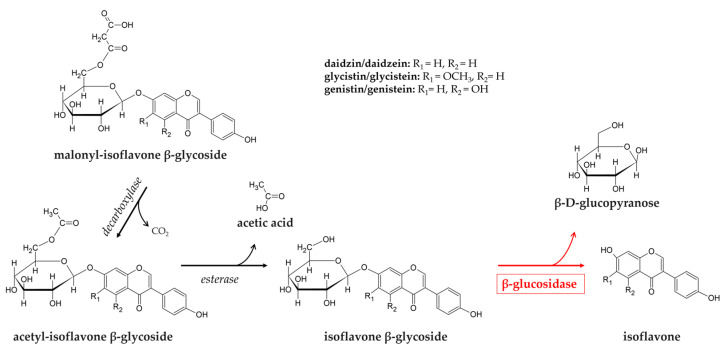
Release of free isoflavone from the three main soy isoflavones glycosides and their acetylated/malonylated forms. Representation of the two-step process by which the acetyl- and malonyl-forms of soy-glycosylated isofavones (daidzin, glycystin, and genistin) are hydrolyzed by esterase and β-glucosidase to release the free form of isoflavone (daidzein, glycistein, and genistein).

Soybeans (*Glycine max*) are important polyphenol sources in the diet because of their high levels of isoflavones [107]. Major isoflavones in soybean consist of three aglycones (daidzein, glycitein, and genistein) and their β-glycosides (daidzin, glycitin, and genistin), and acetyl and malonyl-conjugated β-glycosides (6″-O-acetyl daidzin, acetyl glycitin, and acetyl genistin; 6″O-malonyl daidzin, malonyl glycitin, and malonyl genistin) [108]. The O-β-glycosidic bonds of isoflavones are partially hydrolyzed in the gut primarily by microbial β-glycosidases to their aglycones, daidzein, genistein, and glycitein (Figure 2). Glucoside isoflavones are very poorly absorbed in the small intestine as compared with their aglycones, because of their greater molecular weight and higher hydrophilicity of the glucosides [109,110]. Furthermore, the isoflavone glucosides are known to be less bioactive than their respective aglycones [111].

Several studies have demonstrated that the content of aglycones in soy products was increased after microbial fermentation by LAB, which may be due to the changes of β-glucosidase activity [112,113,114]. Therefore, the use of these bacteria as starters, with the aforementioned enzymatic activity, in soy milk fermentation could contribute to increasing bioavailable isoflavones [115,116], thereby increasing the nutritional values and health benefits of fermented soy products [106].

Aglycones’ release is due to β-glucosidase activity on β-glucosides, which in most cases are also present as malonylated and acetylated forms. In these latter cases, β-glucosidase is part of a two-step process, which also requires an esterase enzyme to remove acetylation (Figure 2).

It has been shown that *L. plantarum* LP 95 was able to efficiently bio-transform glycosides to their bioactive aglycones, which could thus be used as a functional starter culture to increase the antioxidant activity of the fermented soymilk products [37,81]. Other studies have confirmed that several *L. plantarum* strains have great potential to enrich bioactive isoflavones in fermented soy milk products [115,117,118,119]. In recent studies, it has been shown that soy milk fermented from *L. plantarum* 200655 and *L. plantarum* KU210152 can be used as a prophylactic functional food with neuroprotective effects against oxidative stress [120,121]. Consistently, another study highlighted the increased antioxidant capacity of the *L. plantarum*-Y16-fermented soybean milk, with respect to an unfermented one, whose ethanol and water extracts were able to protect HepG2 cells against ABAP oxidative damage; this was reported to be dependent on the activation of the Nrf2/Keap1 signaling pathway and the upregulation of the expression of antioxidant systems such as heme oxygenase-1, superoxide dismutase, catalase, and glutathione peroxidase [122].

Therefore, increased availability of aglycones found in soy milk fermented with *L. plantarum* may be useful for designing new functional foods.

Moreover, use of selected *L. plantarum* strains which are more effective in increasing product bioactivity can also significantly increase the quality of a soy-waste product as okara. Ultrasonic treatment of *L. plantarum* BCRC 10357 was applied to induce a biological stress response resulting in a 100% increase in β-glucosidase activity, with this later responsible for the biotransformation of isoflavone glycosides to bioactive aglycones (daidzein and genistein) in okara [123]. In addition, the fermentation of enzymatically hydrolyzed okara by a *L. plantarum* UFG169 strain was reported to increase the content of both aglycone isoflavones and vitamin B2, as well as a reduction in off-flavors, thus improving both the nutrition and digestibility of this product [124].

### 2.2. Cassava

Cassava (*Manihot esculenta* Crantz), also known as yucca, manioc, or mandioca, is a perennial and herbaceous shrub that belongs to the class Malpighiales and Family Euphorbiceae. This crop has great social value and cultural identity, and it is now extensively cultivated throughout tropical and subtropical regions, mainly for its edible tubers as a source of carbohydrates, flavonoids, fiber, vitamin C, and minerals [125,126,127,128].

According to the Food and Agriculture Organization (FAO), cassava ranks fourth as a food crop in the developing countries, after rice, maize, and wheat [129]. Despite the advantages coming from its starchy tubers, other organs of cassava plant, such as leaves, can be also used for edible purposes. However, these less notable parts are characterized by a low protein content, rapid post-harvest deterioration, and the presence of cyanogenic glucosides, which are seen as major drawbacks which strongly limit its utilization as a food [130].

**Figure 3:**
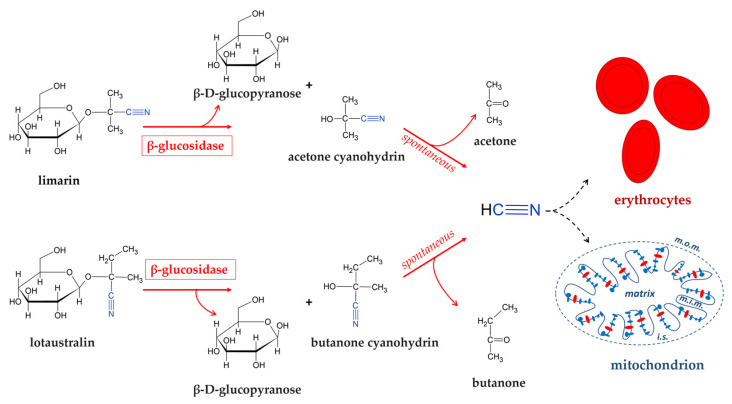
Cyanidric acid generation by cyanogenic glycosides present in cassava tissues. Degradation of the two principal cyanogenic glycosides (limarin and lotaustralin) of cassava by β-glucosidase activity with production of glucose and subsequent release of cyanidric acid. Targets of this high toxic compound are haemoglobin of erythocytes and cytochrome c oxidase, the complex IV (in red) of the respiratory chain embedded in the inner membrane of the mitochondrion. Abbreviations: m.o.m., mitochondrial outer membrane; i.s., intermembrane space; m.i.m., mitochondrial inner membrane.

Consumption of improperly processed cassava may constitute a health problem in rural areas of sub-Saharan African countries where cassava-derived products provide a high percentage of the daily calory intake [131]. In severe cases, this may result in acute cyanide intoxication and in chronic paralytic diseases such as konzo and neurological disorders [132,133]. Moreover, cyanogenic glucosides are spread in all parts of the cassava plant, with the highest amounts in the leaves and the root cortex (skin layer) and are present in bound form, mostly 2-(β-D-glucopyranosyloxyl)isobutyronitrile (linamarin) and, to a lesser extent, its derivative 2-(β-D-glucopyranosyloxyl)methylbutyronitrile (lotaustralin) (Figure 3) [134].

These cyanogenic glucosides are not toxic as such because they are absorbed in the gastrointestinal tract and eliminated as such through urination. However, cyanogenic glycosides are hydrolyzed into acetone cyanohydrin by the glycosidases of gut microbiota. The acetone cyanohydrin was degraded spontaneously in the small intestine in which it had alkaline pH conditions. This degradation releases hydrocyanic acid (HCN), which bound to methemoglobin (Figure 3) [135,136] and, as known, exerts its toxicity by inhibiting the cytochrome oxidase, the complex IV of mitochondrial respiratory chain, thus preventing cellular utilization of oxygen [137]. The presence of these cyanogenic glucosides is the major limiting factor to direct utilization, thereby necessitating its processing prior to consumption. The introduction of new processing methods has helped to reduce cassava’s cyanogenic content and, therefore, exposure levels to its cyanogenic compounds. Cassava is traditionally processed by a wide range of methods, such as boiling, roasting, drying, cold water leaching, or fermentation [127], which reduce its toxicity. Thus, during fermentation, the roots are softened, and there is a disintegration of the tissue structure which causes linamarin to come into contact with endogenous linamerase, which is found in the cell wall, and microbial linamerase. These enzymatic activities result in subsequent hydrolysis into glucose and acetone cyanohydrin which are easily broken down into acetone and HCN. During the natural drying phase, free HCN evaporates easily by having a boiling point of 26 °C.

However, in spontaneous cassava fermentation, the activity of β-glycosidase is often not sufficient to break down all cyanogenic glycosides [138]. Moreover, the linamarase elaborated by both cassava plant tissues and fermenting microorganisms has been found to be unstable under the high acidic conditions characteristic of the latter part of natural fermentation. Therefore, it is important to review the detoxification methods of cassava and improve their effectiveness for greater consumption of cassava-based foods [139].

The cassava fermentation process can be carried out naturally (spontaneous fermentation) by relying on the native microbial populations present in the raw materials and in the environment.

However, the wide range and complexity of the microbiota of spontaneous cassava fermentation are the main factors responsible for the lack of homogeneity and low product quality [140]. The use of exogenous β-glucosidases from microbial sources is suggested, which hydrolyze these cyanogenic glycosides at an elevated level [141,142].

*L. plantarum* and other lactic acid bacteria (LAB) have been reported as the prevalent microorganisms associated with the spontaneous fermentation of cassava [128,143,144,145,146,147,148,149,150]. Some studies have shown that it is possible to significantly degrade cyanogenic glycosides and reduce free HCN in cassava through fermentation using *L. plantarum* as a single starter or in co-culture with other microorganisms [99,101,140,150,151,152,153,154,155].

Therefore, further studies are desirable for the establishment of new starter cultures that can contribute to the standardization of cassava fermentation conditions, thus ensuring higher quality products and consumer acceptability.

Especially, the selection and use of *L. plantarum* as a starter may be an effective biotechnological strategy that may allow for greater preservation, flavor enhancement, cyanide reduction, and improved functional properties of fermented cassava-based products [127].

### 2.3. Olive

Another emerging field of interest for *L. plantarum* β-glucosidases application is represented by olive production. One of the main problems of the olive industry, in fact, is the bitterness of olives which is principally due to the main representative of olive polyphenol glucosides, namely oleuropein [156]. Oleuropein is an O-glycosylated compound constituted by a D-glucose β (1-4) bound to aglycone, which can be hydrolyzed by the β-glucosidase enzyme resulting in D-glucose and aglycone production [157]. At present, the widely used method to debitter olive consists in the alkalyne treatment of drupes by means of NaOH solution; however, this method poses a series of concerns related to both the consumers (treatment-dependent reduction in nutrients) and the environment (wastewaters entrenched in toxic NaOH) [158]. At the same time, the olive debittering represents a committed step in the production of table olives giving rise to the search for alternative NaOH-free methods [159]; in this regard, the activity of microbial β-glucosidases proved to be able to hydrolyze oleuropein, thus producing low-molecular-weight phenolic compounds such as hydroxytyrosol and tyrosol [85,160]. Moreover, several papers showed the capability of *L. plantarum* to hydrolyze oleuropein, as well as the occurrence of this microorganism among the spontaneously fermenting species of table olives [45,161,162]. For these reasons, several strains of *L. plantarum* have been proposed as microbial cultures for table olive fermentation, since their adaptability to fermentation conditions, as well as high β-glucosidase activity makes this species particularly useful in olive debittering [87,163,164,165,166]. More recently, an elegant study [167] proposed three different mechanisms for the conversion of oleuropein into the active compound hydroxytyrosol, which seems to depend on the *L. plantarum* strain and need, besides β-glucosidases, and also the action of esterase activities.

In addition to olive debittering, *L. plantarum* fermentation has been suggested as a potential approach also for the recovery of valuable bioactive compounds, as hydroxytyrosol and tyrosol, from olive mill wastewater. This species, in fact, shares with the yest *Wickerhamomyces anomalus* the ability to increase the content of hydroxytyrosol in wastewater phenolic extract, with both microorganisms proving to be more efficient than the commercial enzyme in 2 h bioconversion tests [168]. In addition, a very recent study showed other functional properties of *L. plantarum* present in olive mill wastewater, such as the notable acidification capability and the production of antibacterial compounds [169]. These results provide strong evidence in making this species a candidate and its β-glucosidase activity as a powerful tool in the management of waste and by-products from the olive industry.

## 3. Fermented Beverages

### 3.1. Alcoholic Beverages

#### 3.1.1. Wine

The wine LAB, naturally present in grape juice, play a significant role in winemaking by guiding a biological process known as malolactic fermentation (MLF).

This process involves the conversion of L-malic acid to L-lactic acid via malate decarboxylase, resulting in a reduction in wine acidity, providing microbiological stabilization and modifications of wine aroma [170].

In the last decades, various papers have shown that LAB metabolism also involves a large array of secondary enzymatic activities capable of generating many volatile secondary compounds as reviewed by Virdis and collaborators [171]. Many studies have shown that some non-*Saccharomyces* yeasts with high β-glucosidase activity play a vital role in improving the aroma complexity of wines by releasing aroma compounds from glycosidic precursors during fermentation [31,32,33]. However, several studies have demonstrated the presence of β-glycosidase activity in wine LAB, leading to the release of free volatile compounds as terpenes [172,173].

*Oenococcus oeni* is the main bacterial species responsible for malolactic fermentation; however, in the last two decades, it has been highlighted that other LAB associated with MLF have enormous potential to influence the composition of wine [171,174].

Among all the species, *L. plantarum* is frequently found on grapes and in wine and is considered as a new generation of MLF starter due to its ability of high ethanol tolerance and good enological characteristics [44,175,176,177,178,179,180,181,182,183]. In addition, L. *plantarum* has a wide range of enzymes, including β-glucosidase, which can also contribute significantly to the formation of wine aroma during the winemaking process [33,55,94,184].

It is because of these characteristics that some commercial starters belonging to *L. plantarum* species have been released in the last decade [185].

The hydrolysis of glycosides, previously reported during the malolactic fermentation through selected *L. plantarum* strains, may be considered as an interesting option to improve the sensorial characteristics of the wines.

Iorizzo et al. highlighted that some L. *plantarum* strains, candidates for MLF, were able to release specific terpenes from odourless grape glycosidic precursors [94]. In another study, *L. plantarum* M10, used as a malolactic starter after the alcoholic fermentation of Fiano grape juice, caused a significantly higher concentration of linalool in the wine [183].

Other authors found a significant increase in β-citronellol and 2-phenylethyl alcohol amounts after MLF with *L. plantarum* UNQLp 11 [186]. β-Citronellol is an alcoholic monoterpene that is most abundant in musts and wines and is often found as an odourless glyco-conjugated compound [65]. In addition, it has been hypothesized that β-glucosidase activity could also explain the increase in 2-phenylethyl alcohol (an aromatic alcohol that contributes to sweet floral attributes) in wine fermented with UNQLp 11, as previously described for other LAB strains [88].

In another study, the β-glucosidase activity of *L. plantarum* USC1 was stable between pH 4.5 and 7.5 and with a maximum activity at pH 5.0 and was completely inactivated at pH values below 4.0. The optimum temperature was 45 °C, and the enzyme was active against a wide range of aryl b-glucosides and b-linked disaccharides [187].

Brizuela et al. analyzed the amount of 1-octanol (mg/mL) obtained by the hydrolysis of the precursor octyl β-D-glucopyranoside in sterile Pinot Noir wine containing 14.5% *v*/*v* of ethanol, at different pH values (3.2, 3.5, and 3.8); the results of this study showed that the activity of β-glucosidase is reduced at low pH, but induced in the presence of high ethanol content [188].

Several studies have demonstrated that the β-glucosidase activity is mainly affected by pH, temperature, ethanol, and sugars [189,190,191,192].

Therefore, screening of *L. plantarum* strains for their glycosidase activities is important and should be performed based on the substrate to be fermented [88,193,194,195].

#### 3.1.2. Beer

Sour beer is traditionally produced through spontaneous fermentations, involving complex microbial consortia, and is characterized by higher concentrations of organic acids. While the production of conventional beer is usually limited to yeast fermentation, the traditional production methods for sour beer, such as Lambic and Geuze beers, originating from Belgium, involve a spontaneous fermentation by multiple microorganisms, including yeasts and bacteria [196,197,198,199,200].

Interest in sour beer has increased substantially in recent decades, and research is underway on both spontaneous fermentations and alternative production techniques [201]. Pure-culture fermentations with strains of *L. plantarum* and *Saccharomyces cerevisiae*, in conjunction with the careful application of processing steps, offer a valid alternative to facilitate the production of sour beer. This approach provides a higher level of process control and more rapid fermentation compared to traditional methods [202,203,204].

The phenolic compounds found in wort and beer, especially phenolic acids and flavonoids, are derived mainly from barley malt and hops and are often present as glycosides [205]. The β-glucosidase activity promotes the bioavailability of these compounds by releasing the aglycones. These phenolic compounds have several functional properties in beer, influencing its colloidal stability, flavor, color, and after deglycosylation increase some beneficial biological effects on human health [206,207,208].

In a recent study, the co-inoculation of *L. plantarum* CECT 9567 and *S. cerevisiae* was applied for the production of a probiotic beer [209]. The authors attribute the higher polyphenol content observed in beers brewed with co-inoculation to two phenomena: hydrolysis of bound polyphenols and increased free polyphenols. These phenomena are significantly related to β-glucosidase activity [210,211].

### 3.2. Non-Alcoholic Fermented Fruit Products

Plant-based foods, including fruits and vegetables, are naturally rich in minerals, vitamins, dietary fibers, antioxidants, and many other beneficial nutrients that make them essential components of a healthy and balanced diet. Due to new healthy trends, consumption of fruit and vegetable juices have increased in recent years [212].

Two predominant fermentation pathways have been identified in the production of fruit juices: the alcoholic pathway, which involves the utilization of yeast, and the non-alcoholic pathway, which relies on the action of LAB.

Being a traditional food biotechnology, fermentation by LAB is widely used for fruit and vegetable fermentation to convert bioactive components, enhance beneficial properties, extend shelf-life, and improve sensory characteristics of final products [213,214].

Fermentation by LAB increases the content of functional nutrients, including polyphenols, flavonoids, organic acids, polysaccharides, amino acids, vitamins, minerals, and other efficacious components, giving the fruit excellent antioxidant, antibacterial, anti-inflammatory, and gut microbiota modulation activities [215,216,217].

Moreover, LAB fermentation can impart distinctive fruity and floral aromas to fruits through the production of esters, ketones, alcohols, terpenes, etc. [218,219,220].

Among the LAB, *L. plantarum* is quite interesting, as far as its application in the fermentation of a wide range of plant-based substrates is concerned, such as vegetables and fruit juices, since it has genome plasticity and high versatility and flexibility [213].

Several studies have shown that *L. plantarum*, used as a starter culture, facilitates the enhancement of flavor and aroma in fermented fruit juices through its β-glucosidase activity. Pomegranate juices fermented by *L. plantarum* POM1 and LP09 were characterized by high levels of terpenes, such as limonene, β-myrcene, γ-terpinene, α-terpinene, α-terpinolene, and p-cymene [97]. Monoterpenes are present in pomegranate juice as either free or glycosidically conjugated precursors and the release of glycosidically bound aromatic compounds has been shown to result in the modification or enhancement of its characteristic flavor [221]. It has been found that Sabre mango juice fermented with *L. plantarum* L75 produced higher levels of β-myrcene [222]. A similar increase in β-myrcene was reported in *L. plantarum* POM1- and LP09-fermented pomegranate juices [97].

In addition to the flavor properties, several biological activities were recognized from β-myrcene as having anxiolytic, antioxidant, anti-aging, anti-inflammatory, and analgesic effects in mammals [223], as well as toxicity against pest [224].

A recent study showed that the nutritional quality and flavor characteristics of apricot juice can be improved by *L. plantarum* LP56 fermentation; specifically, after 6 h, there was a significant increase in the content of volatile compounds, including α-terpineol, nerol, β-pinene, and terpinene, reaching its maximum level [225].

Myrtenol is a volatile compound belonging to the terpenoid family of monocyclic monoterpenes and contributed to the woody, pine, balsam, sweet, and mint notes. In addition, several reports demonstrated the pharmacological properties of myrtenol, including its antioxidant, antibacterial, antifungal, antidiabetic, anxiolytic, and gastroprotective activities [226]. Fermentation of Momordica charantia juice by *L. plantarum* NCU116 improved its aroma profile, with myrthenol as the main aromatic compound, and resulted in a beneficial effect on the physicochemical properties, bioactive compounds, and antioxidant property of the juice [227].

Ricci et al. detected, among the terpene and norisoprenoid class, an increase in limonene, β-linalool, β-damascenon, and eugenol in elderberry juice fermented by *L. plantarum* 285 [228].

The increase in these compounds could be related to the ability of *L. plantarum* to produce β-glucosidase [94,97,219].

Fruit beverages fermented by *L. plantarum*, not only are characterized by a pleasant aroma and taste, but show many health-promoting benefits due to their content of metabolites such as vitamins, organic acids, and phenolic compounds [229,230,231,232].

Several studies have shown that the bioavailability of phenolic compounds is enhanced by different LAB after fermentation of different fruit products [218,233,234,235].

According to several studies, *L. plantarum* produces enzymes such as β-glucosidase during the fermentation process, which is able to hydrolyze phenolic glycosides to the corresponding aglycones, which have radical scavenging properties [211,236,237]. This process results in an increase in the antioxidant activity of the fermented product [238].

The antioxidant activity of phenolics is related to their chemical structure. In general, flavonoid compounds present a stronger antioxidant activity than non-flavonoids, and combined forms such as glycosides present a lower activity than the free forms [239].

In a study conducted by Meng et al. the effect of different *L. plantarum* strains on the physicochemical characteristics and antioxidant activities of loquat juice was investigated. Results showed that nerolidol production was significantly upregulated in loquat juices fermented by *L. plantarum* LP2 [240].

Nerolidol, a terpenoid has good anti-inflammatory, antioxidant, neuroprotective, and cardioprotective activities [241,242]. Furthermore, after fermentation by *L. plantarum* LP2 the antioxidant activity and the total flavonoid content in loquat juice significantly increased.

Landete et al. showed that deglycosylation by *L. plantarum* CECT 748 transformed food aryl glycosides (phloridzin, esculin, daidzin, and salicin) into their corresponding aryl aglycones (phloretin, esculetin, daidzein, and saligenin). Therefore, in addition to the improvement of their bioavailability, the deglycosylation of specific aryl glycosides by *L. plantarum* CECT 748 increase the antioxidant activity of glycosylated phenolic compounds [194]. Moreover, besides the aglycone release, the antioxidant activity in fermented foods can be modulated also by other mechanisms, as antioxidant enzymes, bioactive peptides, and exopolysaccharides produced by *L. plantarum* [243].

Table 2 shows the main positive effects of the enzymatic activity of *L. plantarum*, mainly attributable to β-glucosidase, in different fermented fruit products. The data refer to articles published in the last 10 years. However, the impact of fermentation on phenolic compounds seems to depend heavily on the bacterial strain used and the starting material.

Li et al. reported that LAB fermentation with *L. plantarum* 90 significantly increased the total phenolic content, while decreasing the total flavonoid content in fermented jujube juices [254].

The same author had found that the fermentation of apple juice by *L. plantarum* ATCC14917 caused an increase in antioxidant activity while decreasing the total content of phenols and flavonoids [244], attributing to, according to Tian, the greater antioxidant captivity detected in other possible mechanisms [267].

In another study, the flavonol glycosides in sea buckthorn as well as anthocyanins in chokeberry remained unaffected by the fermentation with several *L. plantarum* strains obtained from DSMZ (Braunschweig, Germany) [268].

Wei et al. [269] reported a general decrease in anthocyanins, phenolic acids, flavonols, and flavanols in bog bilberry juice fermented with *L. plantarum* B7 or *L. plantarum* C8-1.

Therefore, this suggests that careful selection within the *L. plantarum* species is crucial in order to identify the most suitable strains to be used for each specific biotechnological application aimed at improving the functional properties of the final products [270].

## 4. Conclusions

In the last decades, the needs of both producers and consumers in the food sector have continuously grown, thus requiring a particular attention not only to organoleptic aspects but also in terms of health and well-being. This review shows how the enzyme β-glucosidase can be considered crucial for the hydrolysis of several glycosides that give added value to the fermented food matrix. In particular, the activity of β-glucosidase during fermentation by *L. plantarum* can be considered an important biotechnological strategy in order to increase the nutritional, sensory, and functional properties of specific fermented foods. The studies cited in this review showed that the optimal conditions for the β-glucosidase activity differs extensively among the *L. plantarum* strains and is significantly affected by substrate composition and culture conditions. Therefore, it is essential to optimize these conditions to improve this enzymatic activity and also, according to the production process adopted, to obtain each specific fermented food. Considering that lactic fermentation is an important technology to increase functional properties of fermented foods, we believe that the selection of β-glucosidase-producing *L. plantarum* strains should remain a focal point of interest in future research, since it can be a valid tool for the design of new functional foods.

## Figures and Tables

**Table 1:** Main effects of the β-glucosidase activity in plant-based products fermented by some LAB and bifidobacteria.

Food	LAB Species	*β*-Glucosidase Function	Refs.
soybean products	*Lactococcus lactis* *Lacticaseibacillus casei* *Limosilactobacillus fermentum* *Limosilactobacillus mucosae* *Enterococcus faecalis* *L. plantarum* *Lacticaseibacillus rhamnosus * *Bifidobacterium pseudocatenulatum* *Bifidobacterium breve*	deglycosylation of isoflavones (genistin, daidzin, and glycitin)	[77,78,79,80,81]
olive	*L. plantarum* *L. casei* *Lacticaseibacillus paracasei* *Bifidobacterium lactis* *Enterococcus faecium* *Lactiplantibacillus pentosus*	debittering (hydrolysis of the oleuropein)	[82,83,84,85,86,87]
alcoholic beverages (wine and beer)	*Oenococcus oeni* *Pediococcus* spp. *L. plantarum*	deglycosylation of flavor precursors with release of free volatile organic compounds	[88,89,90,91,92,93,94]
non-alcoholic fermented fruit	*L. rhamnosus* *L. plantarum* *Leuconostoc mesenteroides* *Levilactobacillus brevis* *Bifidobacterium* spp.	deglycosylation of flavor precursors with release of free volatile organic compounds	[82,95,96,97]
cassava	*L. plantarum* *L. mesenteroides*	hydrolysis of cyanogenic glycosides (linamarin)	[98,99,100,101]

**Table 2:** Main positive effects potentially related to β-glucosidase activity of *L. plantarum* in fermented fruit.

Fruit Processed	Product Type	*L. plantarum* (*Lp*) Strains	Main Positive Effects	Ref.
Apple	Juice fermented at 37 °C for 72 h.	*Lp* ATCC14917	Increased antioxidant activity and decreased total phenolics and flavonoid content.	[244]
Apple	Juice fermented at 37 °C for 80 h	*Lp* ST-III	Improved flavor profile	[245]
Apple	Single juices from nine apple cultivars fermented at 37 °C for 24 h	*Lp* CICC21805	Increased terpenes D-limonene and eugenol in some apple cultivars	[246]
Apricot	Juice fermented at 37 °C for 12 h	*Lp* LP56	Increased antioxidant activity and total phenolics; improved flavor profile	[225]
Bergamot (Citrus Bergamia Risso)	Juice fermented at 37 °C for 72 h	Single and mixed starter: *Lp* PTCC 1896 *Lp* AF1 *Lp* LP3	Increased antioxidant activity	[247]
Buckthorn berries (*Hippophaë rhamnoides* L.)	Juice fermented at 30 °C for 72 h	*Lp* DSM 10492 *Lp* DSM 20174 *Lp* DSM 6872	Increased antioxidant activity and flavonoids	[248]
Cactus (*Opuntia ficus-indica* L.)	Cladodes pulp fermented at 30 °C for 24 h	Single starters: *Lp* CIL6 *Lp* POM1 *Lp* 1MR20	Increased antioxidant activity and flavonoids (kaemferol and isorhamnetin)	[249]
Cherries (*Prunus avium* L.)	Juice fermented at 37 °C for 48 h	*Lp* JYLP-375	Improved flavor profile	[250]
Cranberrybush/Gilaburu (*Viburnum opulus* L.)	Juice fermented at 30 °C for 12 days	*Lp*-23	Increased antioxidant activity and terpenes	[218]
Elderberry (*Sambucus nigra* L.)	Juice fermented at 37 °C for 48 h	Single starters: *Lp* POM1 *Lp* 1LE1 *Lp* C1 *Lp* 1486 *Lp* 285	Increase in terpenes and norisoprenoids (limonene, β-linalool, β-damascenone, and eugenol)	[228]
Grapes	Juice fermented at 37 °C for 32 h	Single and mixed starter: *Lp* 90 *L. casei*	Increased total phenolics and improved flavor profile	[251]
Hawthorn (*Crataegus pinnatifida*)	Pulp fermented at 37 °C for 12 h	Mixed starter: *Lp*, *Lactobacillus acidophilus* and *L. casei*	Increased total phenolics and flavonoids	[252]
Jujube (*Ziziphus jujuba* Milll.)	Pulp fermented at 37 °C for 24 h	*Lp* CICC 20265	Improved flavor profile	[253]
Jujube (*Zizyphus jujuba* Mill.)	Juice fermented at 37 °C for 48 h	*Lp* 90	Increased antioxidant activity and flavor profile	[254]
Jujube (*Ziziphus jujuba* Milll.)	Juice fermented at 37 °C for 28 h	Single and mixed starter: *L. rhamnosus* GG *Lp*-1 *Lp*-2 *L. paracasei* 22709 *Leuconostoc mesenteroides* 22264	Decreased total phenolics and increased total flavonoid content; improved flavor profile	[255]
Lemon (*Citrus limetta*)	Juice fermented at 37 °C for 48 h	*Lp* LS5	Increased antioxidant activity	[232]
Litchi (*Litchi chinensis* Sonn.	Juice fermented at 37 °C for 40 h	Single starters: *Lp* LP28 *Lp* LP226 *Lp* LPC2W	Increased terpenes citronellol, linalool, geraniol, and prenol	[256]
Loquat (*Eriobotrya japonica* Lindl.)	Juice fermented at 36 °C for 48 h	*Lp* LZ 2-2	Increased antioxidant activity, total phenolics, and total flavonoids	[240]
Mango (*Mangifera indica* L.)	Juice fermented at 37 °C for 48 h	*Lp* NCU116	Increased antioxidant activity and total phenolics	[257]
Mango (*Mangifera indica* L.)	Juice fermented at 30 °C for 72 h	Single and mixed starter *Lp L75* *Leuconostoc pseudomesenteroides* L 56	Increased antioxidant activity and improved flavor profile	[222]
Mixed berry (acai berry, aronia, cranberry)	Juice fermented at 37 °C for 36 h	*Lp* LP-115	Increased antioxidant activity	[258]
*Momordica charantia* L.	Juice fermented at 37 °C for 48 h	*Lp* NCU116	Increased antioxidant activity, total phenolics, and total flavonoids	[227]
Mulberry (*Morus nigra*)	Juice fermented at 37 °C for 36 h	*Lp* ATCC SD5209	Increased antioxidant activity and phenolics (phenolic acids, anthocyanins, and flavonols)	[259]
Mulberry (*Morus nigra*)	Juice fermented at 37 °C for 7 days	*Lp* CICC 20265	Increased antioxidant activity	[236]
Mulberry (*Morus nigra*)	Juice fermented at 37 °C for 48 h	*Lp* (single colture and/or in co-colture with other LAB)	Improvement of both nutritional and aromatic profile	[260]
Orange	Juice-milk fermented at 37 °C for 72 h	Single starters: *Lp* TR-7 *Lp* TR-71 *Lp* TR-14	Increased antioxidant activity and total phenolics	[261]
Orange, lemon, celery and carrot	Mixed vegetable juice fermented at 37 °C for 24 h	*Lp* HFC8	Increased antioxidant activity and phenolics (flavonoids and anthocyanins)	[262]
Passion fruit (*Passiflora edulis*), acerola (*Malpighia emarginata*), and jelly palm (*Butia capitata*)	Juice fermented at 37 °C for 24 h	*Lp* CCMA 0743	Increased flavonoids	[263]
Pomegranate (*Punica granatum* L.)	Juice fermented at 30 °C for 24 h	*Lp* ATCC 14917	Increased antioxidant activity and total phenolics	[264]
Pomegranate (*Punica granatum* L.)	Juice fermented at 30 °C for 120 h	Single starter: *Lp* C2 *Lp* POM1	Improved flavor profile	[97]
Sohiong (*Prunus nepalensis*)	Juice fermented at 37 °C for 72 h	*Lp* MCC 297	Increased antioxidant activity, total phenolics, and anthocyanins	[265]
Wolfberry	Juice fermented at 37 °C for 48 h	*Lp* NCU137	Increased antioxidant activity and free phenolics	[266]

## Data Availability

The original contributions presented in the study are included in the article, and further inquiries can be directed to the corresponding author.
